# Leveraging natural language processing, machine learning, and a knowledge-grounded language model to detect linguistic markers of deception

**DOI:** 10.3389/frai.2026.1965099

**Published:** 2026-10-28

**Authors:** Kacey Miller, Andy Chu, Ameeta Reed, Zachary McCord, Ethan Argentine, Jamari Allen, Patti Coggins, Dean Pollina, Stuart Senter, Tarek Shazly, Mark Uline, Nicholas D. Boltin

**Affiliations:** 1 Department of Biomedical Engineering, Molinaroli College of Engineering and Computing, University of South Carolina, Columbia, SC, United States; 2 Molinaroli College of Engineering and Computing, Acute Stress Center, University of South Carolina, Columbia, SC, United States; 3 EntigenLogic LLC, Schaumburg, IL, United States; 4 U.S. Defense Counterintelligence and Security Agency, Cayce, SC, United States; 5 National Center for Credibility Assessment, Columbia, SC, United States; 6 Molinaroli College of Engineering and Computing, Integrated Information Technology, University of South Carolina, Columbia, SC, United States

**Keywords:** credibility assessment, deception detection, natural language processing, machine learning, retrieval-augmented generation, linguistic analysis, psycholinguistics

## Abstract

**Introduction:**

Deception elicits measurable cognitive and linguistic changes, yet accurately identifying deceptive language in high-stakes environments remains a persistent challenge. Although natural language processing (NLP) and machine learning (ML) techniques have shown promise in detecting misinformation and low-stakes deception, their performance on spontaneous, stress-induced speech remains poorly understood. This study investigates linguistic markers of deception and evaluates the performance of supervised ML models and a knowledge-grounded language model, FranklinAsk™, across both synthetic and real-world datasets.

**Methods:**

Linguistic analyses were conducted on responses from a National Center for Credibility Assessment (NCCA) military training exercise involving truthful and deceptive participants. A synthetic dataset was generated using MOSTLYAI to provide a balanced corpus for ML training. Linguistic features were extracted using pretrained NLP models, and supervised classifiers, including logistic regression, Random Forest, Naïve Bayes, and Support Vector Machine (SVM), were trained using LIWC-22 features with 10-fold cross-validation. The FranklinAsk™ RAG system was evaluated using the same participant responses to compare knowledge-grounded reasoning with statistical learning.

**Results:**

Two linguistic features, cardinal numbers (nominal *p* = 0.041) and particles (nominal *p* = 0.0486), reached the unadjusted significance threshold, each with a moderate effect size (*d* = 0.55); neither remained statistically significant after Benjamini-Hochberg FDR correction (adjusted *p* = 0.668 for both). No significant sentiment differences were observed. On the synthetic dataset, logistic regression performed best (F1 = 0.930; ROC AUC = 0.978), but all supervised models showed substantial performance declines when applied to the real-world dataset. In contrast, FranklinAsk™ achieved the highest F1-score (0.519), highest accuracy (0.487), and second-highest recall (0.700) on the military exercise data.

**Discussion:**

These findings highlight the limited generalizability of synthetic-trained deception models to real-world, high-stakes communication and motivate further investigation of knowledge-grounded language models as an alternative approach when labeled real-world training data are limited. Future research should prioritize expanding ecologically valid datasets and exploring hybrid approaches that combine statistical modeling with knowledge-grounded reasoning.

## 1 Introduction

Deception is a complex phenomenon involving intentional or unintentional attempts to mislead a receiver, and its detection remains a persistent challenge across high-stakes contexts such as intelligence vetting, criminal investigations, and organizational risk assessment. Although deception manifests through behavioral, physiological, and linguistic pathways, the accuracy of human judgment remains low. For instance, trained forensic professionals correctly identify deception only slightly above chance levels (approximately 59%) (Loconte et al., 2025). This gap has spurred interest in automated approaches capable of detecting subtle linguistic regularities that accompany deceptive communication.

Additionally, stress plays a central role in the production of deceptive speech. Deception is widely conceptualized as a social-evaluative threat that elicits measurable physiological and cognitive changes (Poppelaars et al., 2019). Stress-induced elevations in cortisol impair prefrontal cortical processes involved in language generation, working memory, and hierarchical planning (Cook and Wellman, 2004; Sale et al., 2008; Puderbaugh and Emmady, 2023). These neurocognitive changes increase the likelihood of less deliberate, more spontaneous linguistic behavior, creating opportunities for computational methods to identify systematic deviations between truthful and deceptive speech.

Natural Language Processing (NLP) offers a principled framework for quantifying such deviations. Recent advances in syntactic parsing, part-of-speech (POS) tagging, semantic modeling, and sentiment analysis have enabled large-scale evaluation of linguistic markers associated with deceptive behavior. Although NLP has been applied extensively to misinformation and fake news detection (Olaleye et al., 2021; Jiang et al., 2020; Samadi and Momtazi, 2023; Boyd et al., 2022; Dev et al., 2024; Zervopoulos et al., 2020; Himdi et al., 2022), far fewer studies have examined linguistic markers of deception in ecologically valid, high-stakes interpersonal contexts. Even fewer evaluate the performance of modern AI systems, such as Retrieval-Augmented Generation (RAG) architectures, against traditional supervised machine learning classifiers.

The Franklin™ family of large language models, developed by EntigenLogic^®^, represents an emerging class of NLP systems designed for domain-specific reasoning. FranklinAsk™, a RAG-based extension, constrains decision support to a curated knowledge base and may offer advantages in settings that require interpretability and information-grounded decision processes (Arslan et al., 2024). However, no prior study has systematically evaluated the utility of RAG systems for deception detection.

The present study offers three contributions:

1. A psycholinguistic analysis of deceptive vs. truthful speech collected during a National Center for Credibility Assessment (NCCA) military training exercise, examining fine-grained POS distributions and sentiment differences using state-of-the-art pretrained NLP models.
2. A comparative evaluation of four supervised machine learning classifiers (logistic regression, random forest, naïve Bayes, SVM) trained on synthetic data generated using MOSTLYAI and tested on both synthetic and real-world responses.
3. The first empirical assessment of FranklinAsk™ as a deception-classification system, using identical datasets to enable a direct comparison with supervised ML methods.

To our knowledge, no prior work has evaluated a RAG-based system for deception detection or directly compared its performance to supervised ML models under identical conditions. This study further contributes a rare high-stakes deception corpus, military training responses collected under social-evaluative threat, which remains underrepresented in computational deception literature. By integrating statistical linguistics, synthetic data generation, supervised learning, and RAG-based inference, this work provides a holistic assessment of NLP-based deception detection approaches and highlights the methodological challenges inherent to real-world applications.

## 2 Related work

Research on deception detection spans several domains, including misinformation analysis, social media text classification, psycholinguistic profiling, and cognitive approaches to lie detection. Although NLP methods have advanced substantially in these areas, their application to high-stakes, spontaneous, deceptive speech remains limited. The following subsections review the most relevant contributions across three major themes: (1) fake news and misinformation detection, (2) linguistic and psychological markers of deception, and (3) NLP-driven lie detection in naturalistic settings.

### 2.1 NLP for fake news and misinformation detection

Fake news detection has become one of the most active areas of NLP research, largely due to the availability of large, annotated datasets and the social impact of misinformation. Traditional and deep learning approaches have achieved high classification performance by leveraging a diverse set of linguistic and semantic features.

Ensemble and hybrid models have demonstrated exceptional performance, with some studies reporting accuracies exceeding 99% on domain-specific misinformation corpora (Olaleye et al., 2021). Feature-engineered architectures such as convolutional neural networks (CNNs), often combined with contextual embeddings or additional modalities, remain widely used. For example, Samadi and Momtazi (2023) developed an enhanced semantic model that integrates sentiment, named-entity profiles, and topic features for multilingual misinformation classification, achieving strong results across English and Persian datasets (Samadi and Momtazi, 2023). Feature extraction remains a central challenge in this domain. Psycholinguistic tools such as LIWC-22 provide structured lexical categories that have proven useful in differentiating deceptive or manipulative text (Boyd et al., 2022). Dev et al. (2024) combined LIWC features with a hybrid LSTM-CNN model to classify multimodal fake news and demonstrated improved robustness relative to single-architecture models (Dev et al., 2024).

Collectively, research on fake news demonstrates the effectiveness of NLP models in detecting linguistic irregularities associated with deception. However, these datasets typically contain well-structured written text, lack the spontaneity of spoken or stress-induced language, and often revolve around deliberate external misinformation rather than interpersonal deception, thereby limiting their generalizability to credibility-assessment contexts.

### 2.2 Linguistic and psychological markers of deception

A substantial body of research has explored how deception affects linguistic style, cognitive load, and expressivity. Deceptive language often reflects increased cognitive effort, reduced lexical diversity, and shifts in syntactic patterns. Psychological studies emphasize that personality, context, and stress all modulate linguistic behavior. Spitzley et al. (2022) analyzed group conversations. They found that personality traits correlate with variation in linguistic markers such as immediacy, sentiment, and lexical complexity, illustrating that deception-related cues cannot be universally applied without contextual control (Spitzley et al., 2022). Tools such as LIWC, SPLICE, and VADER have played an important role in quantifying these psycholinguistic features.

Similarly, Sarzynska-Wawer et al. (2023) showed that only a small subset of linguistic features (e.g., infinitives) consistently differentiate truthful from deceptive narratives (Sarzynska-Wawer et al., 2023). Their findings echo the broader literature: many linguistic cues exhibit weak effect sizes or are strongly dependent on message context. This highlights the need for domain-specific datasets and statistically rigorous analyses. Other studies demonstrate that negative sentiment or increased negation may be associated with deceptive messaging in online settings (Alonso et al., 2021), but these effects tend to be subtle and dataset-dependent.

Taken together, prior work suggests that while linguistic differences between truthful and deceptive language exist, they are typically small, context-sensitive, and require computational methods capable of detecting fine-grained shifts in syntactic and semantic structure.

### 2.3 NLP-driven lie detection and machine learning approaches

Beyond broad misinformation detection, a growing line of work focuses specifically on classifying interpersonal deceptive statements, often using machine learning and psycholinguistic features. Loconte et al. (2023) demonstrated that fine-tuned large language models (LLMs) can differentiate truthful and deceptive short narratives with moderate accuracy (Loconte et al., 2023). Their findings highlight that linguistic features generalize inconsistently across datasets, underscoring the need for cross-dataset evaluation frameworks such as k-fold validation (Anguita et al., 2012).

Machine learning approaches, including SVM, logistic regression, random forest, and naïve Bayes, remain widely used due to their interpretability and reliability across small to medium datasets. Studies such as Zervopoulos et al. (2020) and Himdi et al. (2022) show that traditional classifiers often perform competitively when paired with strong feature-extraction pipelines, sometimes outperforming deep neural networks on structured text (Zervopoulos et al., 2020; Himdi et al., 2022). More recently, Brzic et al. (2023) applied LIWC-22 features to misinformation and deception datasets and found significant performance variation across domains (Brzic et al., 2023). Their results reinforce that dataset characteristics, topic, context, and linguistic style strongly influence deception classification accuracy.

However, despite these efforts, no prior studies have systematically compared supervised machine learning models with Retrieval-Augmented Generation (RAG) systems, nor have they evaluated such models on spontaneous, high-stakes deception elicited under stress. Furthermore, most existing datasets are either crowdsourced, synthetic, or derived from low-stakes laboratory tasks, leaving a gap in real-world applicability.

## 3 Methodology

### 3.1 Data sources

This study used two complementary datasets to examine linguistic markers of deception and to evaluate multiple NLP-based classification approaches. The first dataset consisted of written responses collected during a 2019 NCCA military training exercise, providing naturally occurring high-stakes deceptive and truthful language. The second dataset was a synthetic corpus generated using MOSTLYAI, intended to address the original dataset’s limitations in scale, class balance, and response length.

#### 3.1.1 Military training exercise data

The real-world dataset was collected during a 2019 NCCA mock-sabotage training exercise conducted with U. S. Army basic training soldiers assigned to NCCA who were stationed at the US Army Training Center at Fort Jackson, South Carolina. Participation in the study was voluntary and had no impact on training status. Upon arrival, soldiers were randomly assigned to five-person teams consisting of three truthful “Team Member” participants (Team Alpha) and two deceptive “Sabotage” participants (Team Bravo). Each participant received an individualized mission briefing outlining their assigned role. Team Alpha participants were instructed to work cooperatively to defuse computer-simulated Improvised Explosive Devices (IEDs) and compete against Team Bravo. In contrast, Team Bravo participants were covertly instructed to sabotage the team by misleading others into making incorrect second-button choices during the IED task.

To heighten realism and induce social-evaluative threat (SET), a key mechanism underlying high-stakes deception, participants were told that sabotage was suspected within the group following the mission and that they would undergo an investigative interview to determine whether they had engaged in deceptive behavior. This pre-interview briefing created clear motivational stakes consistent with operational screening contexts.

Following the sabotage exercise, each soldier completed a computer-generated (CG) agent interview, during which they responded to eight open-ended questions used in the present study. The CG agent’s questions were adapted from operational interviewing protocols and were designed to probe perceptions of team integrity, suspicions of sabotage, personal honesty, and descriptions of the participant’s role. All participants, truthful and deceptive alike, received the same set of questions. Soldiers in the sabotage role were explicitly instructed during their mission briefing to lie during the interview when necessary to conceal their Bravo affiliation and maintain the appearance of being a truthful Alpha teammate.

All interviews were audio-captured, and responses were manually transcribed and processed using a custom Python 3.12.7 pipeline. During the interview, participants were given Forced-Choice questions and Open-ended questions. Table 1 contains Open-Ended questions relevant to this study, where the respondent could answer each question thoroughly and in their own words. These transcriptions form the corpus used for linguistic feature extraction, psycholinguistic analysis, and the downstream machine learning and RAG-based deception-classification evaluations described in the following sections. Pretrained HuggingFace models were employed to extract linguistic features relevant to deception detection, including part-of-speech (POS) distributions, syntactic structure, token-level characteristics, and sentiment polarity. These analyses enabled a fine-grained characterization of linguistic differences between truthful and deceptive responses under naturalistic stress.

**Table 1:** Open-ended questions.

Stimulus	Question presented by CG agent
Question 1	If you had to choose someone, which member of Team Alpha do you most suspect and Why?
Question 2	If you had to choose someone, which member of Team Alpha do you least suspect and Why?
Question 3	On a scale of 1 to 5 with 5 being the most honest, how honest are you. Explain your answer.
Question 4	How many members of Team Alpha do you think were secretly working for Team Bravo?
Question 5	When defusing the IED, is it better to stay with the same button on the second press, or switch buttons on the second press? Explain your answer.
Question 6	What would you like to clarify about your involvement in sabotage?
Question 7	Did you ever tell a lie to a person in authority?
Question 8	What have I not already asked you that you would like me to know?

During the interview, participants were allowed to answer each question thoroughly and in their own words.

#### 3.1.2 Synthetic data generation

Because the military dataset was small, unevenly distributed, and often brief, MOSTLYAI was used to generate a synthetic dataset for training supervised machine learning (ML) models. The following prompt was used to generate the synthetic data:

*“A study was conducted at the national center for credibility assessment (NCCA). This study included participants from US Army basic training that gave their consent to participate. Each participant was randomly assigned to one of two groups: Participants in the first group, code-named Alpha, their mission was to work together with the other team members to defuse Improvised Explosive Devices (IEDs) using a computer program that awards one point for each successfully defused IED. It was also explained that they were competing against soldiers on a different team, code-named Bravo. Their goal was to score more points than team Bravo. Team members were told they would be interviewed about their role in the mission following the mission. The other group, the sabotage group, was told they were members of team Bravo. Team members of bravo were told they were enemies of team Alpha and that team Alpha assumes that everyone is on the same team, and that they should not tell anyone that they are a member of team bravo. Team bravo’s goal was to secretly convince the members of team alpha to press the wrong button and explode the simulated IED as many times as they could, by doing this they could score more points than team Alpha. Sabotage participants were told that in their interview following the mission, they must lie when they must and convince the agent that they were members of team Alpha. In the interview following the mission, all participants were asked the same 8 questions:*

*Question 1: If you had to choose someone, which member of Team Alpha do you most suspect and Why?*

*Question 2: If you had to choose someone, which member of Team Alpha do you least suspect and Why?*

*Question 3: On a scale of 1 to 5 with 5 being the most honest, how honest are you?*

*Question 4: How many members of Team Alpha do you think were secretly working for Team Bravo?*

*Question 5: When defusing the IED is it better to stay with the same button on the second press or switch buttons on the second press?*

*Question 6: What would you like to clarify about your involvement in sabotage?*

*Question 7: Did you ever tell a lie to a person in authority?*

*Question 8: What have I not already asked you that you would like me to know?*

*Generate a dataset of 1,000 total responses to each of the 8 questions. Make half of these responses (1–500) truthful, as someone from team alpha was being interviewed. The other half of these responses (501–1,000) should be deceptive, as someone from team bravo (sabotage group) was being interviewed. Each of these responses should be in 2–4 full sentences. They can refer to people by specific names like Brad, Susan, John, etc. Responses to all questions should be in full sentences, including questions 3 and 4. Give me this data in a txt format that can be pasted into an excel file.”*

A total of 1,000 synthetic participants were generated. Each synthetic participant provided responses to all eight interview questions. Consequently, the dataset contained 8,000 individual responses organized into 1,000 participant-level records. Responses across all eight questions were aggregated into a single participant-level record prior to feature extraction, yielding one feature vector per participant and a final classification dataset of 1,000 samples, evenly divided between truthful and deceptive classes. MOSTLYAI’s LLM was pretrained on a broad corpus of scientific and general-domain text, enabling it to generate linguistically coherent responses that reflect patterns found in human narrative and interpersonal communication. Table 2 presents an example of responses generated for each question across the two classes.

**Table 2:** Example responses generated in the synthetic dataset from MOSTLYAI.

Question	Ground Truth = 0 (Truthful)	Ground Truth = 1 (Deceptive)
Q1	I suspect Brad more than others because he often questions protocol and seems tense in covert moves. He has a habit of volunteering to take riskier tasks, which could indicate a need for control. I’d say Brad is my strongest suspicion given his tendency to act first and ask questions later.	I’m most suspicious of Susan because she tends to be silent when issues arise and then provides answers that seem rehearsed.
Q2	I least suspect Susan because she is consistently calm, follows procedures, and helps others stay focused under pressure. Her demeanor suggests reliability, and she rarely pushes for personal credit. Shes the teammate who usually keeps to the plan and communicates clearly.	I least suspect Brad as he is open about his process and does not hide behind vague statements.
Q3	I’d rate my honesty at 4 out of 5. I believe I’m generally forthright, though I sometimes hesitate to share every doubt immediately to avoid causing panic. Overall, I tell the truth as I understand it in the moment.	3. I’m generally honest but I know I sometimes withhold details to avoid unnecessary friction.
Q4	I think maybe 1 or 2 members were secretly aligned with Team Bravo based on inconsistent stories and unexplained lapses in coordination. Its not a large number, but enough to disrupt trust and communication. The pattern seemed to follow certain quieter voices who avoided direct discussion.	I’d guess 1 or 2 members might to be secretly involved with Bravo given the mixed signals in the room.
Q5	On defusing an IED, switching buttons on the second press is safer because it forces verification of a new action and reduces the chance of triggering the device by habit. It teaches discipline to reassess during critical moments.	Switch buttons on second press to prevent predictable patterns and maintain safety.
Q6	I want to clarify that I did not sabotage anything. I’ve been transparent about my role and I’m willing to submit to any review. Any implication otherwise would be a misunderstanding of my actions and intentions.	I’d like to clarify that I’m not involved in sabotage and I would explain any confusion if given the chance.
Q7	Yes, I have told a lie to someone in authority once to protect a colleague from an unfair consequence, but I came clean later. It was a strategic choice under stress, and I’ve learned to avoid it by seeking counsel and being truthful whenever possible.	Yes, I have told a lie to a person in authority before, usually to simplify a complicated situation.
Q8	I would like you to know that I feel a strong responsibility for the teams safety and for maintaining mission integrity. I sometimes feel the burden of suspicion is distributed unevenly, and I’m hopeful we can work on transparency together.	I’d want you to know that I value transparency and loyalty, and I’m willing to discuss any concerns openly.

Ground truth = 0 represents a truthful subject, while ground truth = 1 represents a deceptive subject.

##### 3.1.2.1 Mitigating repetitiveness in synthetic data generation

Data generation was divided into 10 smaller batches of 100 participants each. A default temperature = 1.0 introduced randomness in token selection, while a top-*p* = 0.95 introduced nucleus sampling for diverse and coherent outputs. Each of the 10 batches represented an independent LLM call, ensuring no memory between subsets to minimize repetition and prevent pattern reinforcement. The rich context of the prompt, along with open-ended instructions, afforded creative freedom in LLM generation. Vague guidelines allowed length flexibility (standard deviation = 61.7 characters), indicating healthy variation and natural, human-like verbosity.

The resulting synthetic corpus provided balanced data suitable for supervised model development while preserving the structural format of the original interview task. However, the explicit truthful-versus-deceptive generation instructions may have introduced systematic class-specific linguistic patterns that do not reflect naturally occurring deception.

### 3.2 Data analysis procedures

#### 3.2.1 Statistical analysis of linguistic features

Statistical analyses were performed on the real-world and synthetic datasets to determine whether measurable linguistic differences existed between deceptive and truthful participants. POS tagging used the Flair English model. Thirty-five relevant fine-grained POS tags were retained; tags not relevant to the present analysis (e.g., hyphen, foreign-word, e-mail, and affix tags) were excluded. Eight broader aggregate POS groups and total word count were also computed, yielding 44 extracted linguistic features. In the military dataset, three features (symbols, possessive wh-pronouns, and plural proper nouns) contained zero counts and were excluded from hypothesis testing, leaving 41 tested features. Sentiment was assessed using the Twitter-RoBERTa-base model. Group differences were evaluated using two-sided Welch’s independent *t*-tests with a nominal alpha = 0.05. Benjamini-Hochberg FDR and Bonferroni corrections were applied to account for multiple comparisons, and Cohen’s *d* was used to quantify effect magnitude.

#### 3.2.2 Machine learning classification models

Four supervised ML classifiers, logistic regression, random forest, naive Bayes, and support vector machine (SVM), were evaluated using features derived from LIWC-22. For both datasets, the eight question responses associated with each participant were aggregated into a single participant-level record before feature extraction, yielding one feature vector per participant. A 70/30 train-test split was applied to the synthetic dataset at the participant level, so all eight responses from a given synthetic participant remained in the same partition. Ten-fold cross-validation was used within the training set for model development. Each model was then retrained on the full training set (n = 700) and evaluated on the held-out test set (*n* = 300). The trained models were subsequently applied without retraining to the complete military dataset (*n* = 76). Performance was quantified using accuracy, precision, recall, F1-score, and ROC AUC. Uncertainty in model performance was quantified using participant-level bootstrap resampling with 2000 iterations; 95% confidence intervals were calculated from the 2.5th and 97.5th percentile/BCa bootstrap distribution.

#### 3.2.3 Evaluation of FranklinAsk™

To assess the performance of a knowledge-driven NLP system, we evaluated FranklinAsk alongside the supervised models. We constructed a domain-specific knowledge base from peer-reviewed literature on deception, psycholinguistics, and NLP methodologies (*n* = 21), enabling the RAG system to ground its reasoning in curated external sources. During the ingestion stage, Franklin processes each document using its hybrid-RAG pipeline. In standard operation, Franklin first performs semantic chunking, embeds each chunk using a dense retrieval encoder, and simultaneously constructs a graph-based semantic representation (“entigenation”) that links document concepts to the system’s Base + Domain Knowledge (BDK) graph. This dual representation allows Franklin to conduct hybrid retrieval that combines dense-embedding similarity with a proprietary graph-to-graph comparison algorithm.

For the present study, all 21 peer-reviewed deception-related documents were ingested into Franklin. During review of the ingested corpus, nine documents were determined by FranklinAsk to contain all content directly relevant to linguistic deception cues. Subject matter experts in Credibility Assessment independently confirmed these nine documents based on their direct relevance to deception detection in high-stakes interviews. Document selection occurred prior to model evaluation and was independent of outcome analysis. FranklinAsk then generated synopses of the nine relevant documents, and these synopses were concatenated into a single context file. Because the combined text fit entirely within FranklinAsk’s context window, the system did not need to perform chunk-level retrieval, score competing segments, or invoke its hybrid search mechanism during inference. Under these conditions, FranklinAsk operates in a simplified mode. Instead of selecting and ranking context from distributed chunks, it processes the full aggregated document directly as its knowledge source while still leveraging the same underlying LLM component for reasoning.

This configuration does not alter Franklin’s overall architecture. Franklin remains a hybrid-RAG system engineered to support both dense-vector search and graph-based semantic retrieval. Rather, it indicates that, for this dataset, the full set of relevant knowledge can be provided explicitly as a single context block, bypassing the need for retrieval. In larger or more heterogeneous corpora, FranklinAsk would automatically revert to its standard hybrid search process, synthesizing embedding-based similarity scores and graph-comparison scores through reciprocal-rank fusion to construct a context window optimized for the user’s query. Accordingly, RAG refers here to the underlying FranklinAsk architecture; the experimental configuration is more precisely described as simplified knowledge-grounded inference because dynamic retrieval was bypassed during inference. Appendix A contains additional information on FranklinAsk’s methodology and experimental configuration.

The system was prompted to generate a deception likelihood rating on a 1–10 scale. FranklinAsk does not require model training; therefore, the 70% split of the synthetic data used to train the classification models was instead used to determine the optimal threshold for binary classification. From the training dataset, 4.5 was identified as the optimal boundary to maximize each metric; therefore, the deception likelihood was binarized (1–4.5 = non-deceptive; 5–10 = deceptive) for direct comparison with ML classifiers. The performance of FranklinAsk was evaluated on the same 30% test partition used to evaluate the ML models. The same indices were used to ensure an identical evaluation of subsets.

For the military dataset, each participant’s eight responses were entered into FranklinAsk. The same prompt was used to ask the system to generate a deception likelihood rating, and the threshold established using the synthetic data was applied. To directly compare with machine learning classification models, the entire military dataset (*n* = 76) was evaluated, and performance metrics (accuracy, precision, recall, F1-score and ROC AUC) were computed for both datasets.

## 4 Results

### 4.1 Linguistic analysis of military exercise data

Statistical analysis of the real-world military exercise dataset revealed limited but identifiable linguistic differences between deceptive and truthful participants. Welch’s *t*-tests were conducted for all POS categories and total word count. Among the tested military-exercise features, cardinal numbers (nominal *p* = 0.041) and particles (nominal *p* = 0.049) reached the unadjusted significance threshold. However, neither remained significant after Benjamini-Hochberg (B-H) false-discovery-rate (FDR) correction (adjusted *p* = 0.668 for both) or Bonferroni correction (adjusted *p* = 1.000 for both). These findings should therefore be considered exploratory and interpreted cautiously. At the descriptive level, both features occurred more frequently in deceptive responses; however, because neither association survived correction for multiple comparisons, these patterns should not be interpreted as established deception-related differences. All other features were deemed insignificant in this dataset. A summary table of all *p*-values is provided in Table 3, highlighting the relative distribution of statistical significance across POS categories.

**Table 3:** Summary of *p*-values following a series of Welch’s *t*-tests on all 44 POS features extracted from the military exercise data, including FDR and Bonferroni corrections.

Military exercise features	*p*-value	B-H FDR correction	Bonferroni correction
Cardinal numbers	**0.040992055**	0.668136605	1
Particles	**0.04864658**	0.668136605	1
Possessive pronouns	0.073954244	0.668136605	1
Whpronouns	0.092686754	0.668136605	1
Singular proper nouns	0.105185384	0.668136605	1
Interjections	0.124782867	0.668136605	1
Others	0.159568498	0.693213809	1
Possessive endings	0.159568498	0.693213809	1
Whadverbs	0.160822472	0.668136605	1
All adjectives	0.162960148	0.668136605	1
All conjunctions/prepositions	0.208210969	0.668136605	1
Prepositions	0.233134971	0.668136605	1
Superlative adverbs	0.237394782	0.710644305	1
Tos	0.242019123	0.693213809	1
Regular determiners	0.261681172	0.693213809	1
All determiners	0.270522462	0.693213809	1
Superlative adjectives	0.309861226	0.693213809	1
Singular nouns	0.311990183	0.710644305	1
Modal verbs	0.396831267	0.853697829	1
Gerunds	0.416437965	0.853697829	1
Regular adjectives	0.442887996	0.864686087	1
All nouns	0.466948386	0.870221993	1
Total words	0.512093537	0.87289487	1
Coordinating conjunctions	0.574079478	0.87289487	1
Past participle verbs	0.583468618	0.87289487	1
Plural nouns	0.611221261	0.87289487	1
Existential Theres	0.687814995	0.87289487	1
All pronouns	0.698634201	0.87289487	1
Past tense verbs	0.700044397	0.87289487	1
Singular 3rd person verbs	0.707983772	0.87289487	1
Personal pronouns	0.742335332	0.87289487	1
All adverbs	0.759376205	0.87289487	1
Regular adverbs	0.769475326	0.87289487	1
Predeterminers	0.77193143	0.87289487	1
Comparative adjectives	0.78341301	0.87289487	1
Singular Non3rd person verbs	0.791382681	0.87289487	1
Comparative adverbs	0.822759929	0.87289487	1
Whdeterminers	0.830314633	0.87289487	1
Base form verbs	0.899419157	0.921904636	1
All verbs	0.964467484	0.964467484	1

Symbols, possessive whpronouns, and plural proper nouns are exluded from this list because the feature extraction returned a count of zero for each. Bold *p*-values indicate nominal statistical significance (*p* < 0.05) before multiple-comparison correction.

Effect-size analysis further contextualized these findings. Cohen’s *d* indicated moderate unadjusted effects for both cardinal numbers (*d* = 0.55) and particles (*d* = 0.55); because the corresponding hypothesis tests did not remain significant after multiplicity correction, these effects are interpreted as exploratory rather than confirmatory. Figure 1 shows the broader distribution of effect sizes, where features such as wh-pronouns, singular proper nouns, and interjections (*d* ≈ −0.30) showed small effects favoring truthful responses but did not reach statistical significance. Adjectives exhibited a small-to-moderate effect size (*d* = 0.36) but did not reach statistical significance (*p* = 0.163).

**Figure 1:**
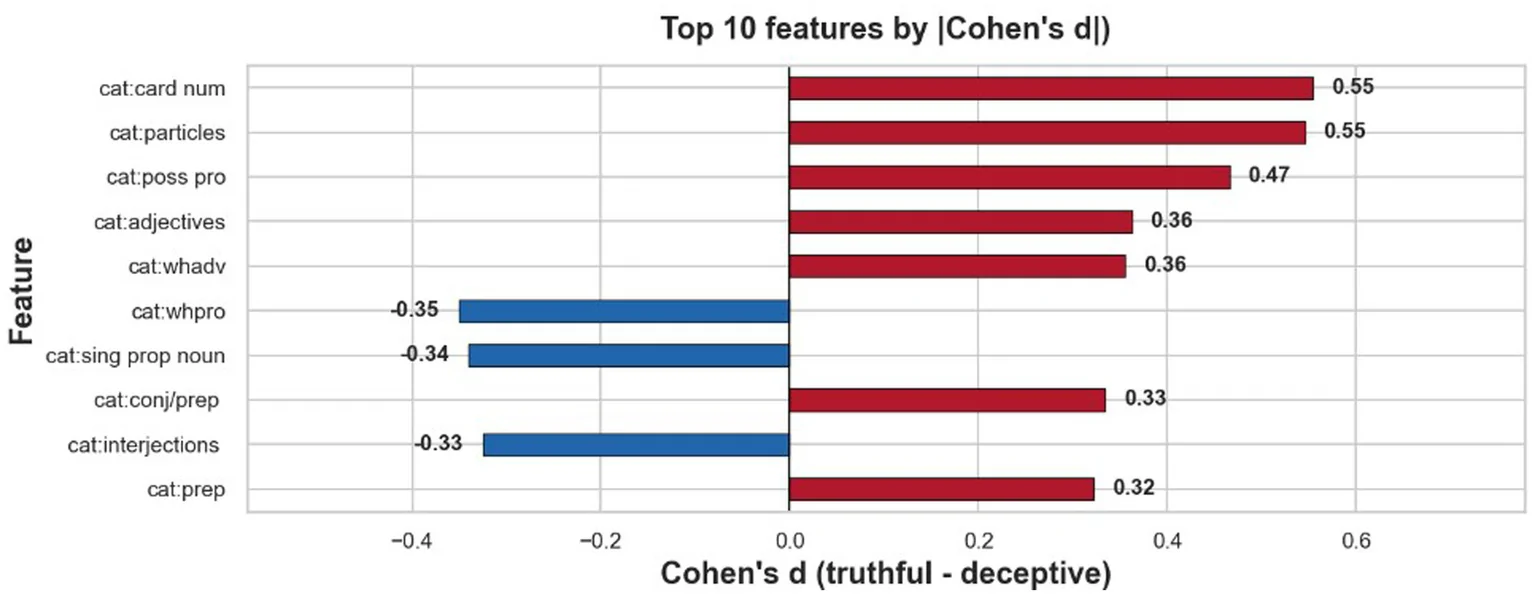
Cohen’s *d* effect sizes for the 10 most discriminative POS categories found in the military exercise dataset. Large absolute values (|d| > 0.8) indicate strong class separation. Negative values (blue) indicate prevalence in truthfulness and positive values (red) indicate usage in deception. Cardinal numbers and particles exhibit moderate effects favoring deception, while wh-pronouns, singular proper nouns, and interjections show minor effects favoring truthful responses.

Sentiment analysis revealed minimal differences across groups. As shown in Table 4, deceptive responses displayed slightly higher negative sentiment (mean = 0.342, SD = 0.063) than truthful responses (mean = 0.331, SD = 0.060), but this difference was not significant (*p* = 0.430, *d* = 0.188). Neutral sentiment was slightly lower in deceptive responses (mean = 0.609) than truthful responses (mean = 0.618), with a nonsignificant effect (*p* = 0.487, *d* = −0.169). Positive sentiment was rare across both groups, showing no meaningful differences (*p* = 0.526, *d* = −0.148). Collectively, these findings show that, within this dataset, sentiment patterns do not meaningfully differentiate deception from truthfulness.

**Table 4:** Sentiment analysis summary for deceptive and non-deceptive respondents found in the military exercise dataset, including group means, standard deviations, Cohen’s *d*, and *p*-values across negative, neutral, and positive polarity.

Sentiment	Deceptive mean	Deceptive SD	Truthful mean	Truthful SD	Cohen’s *d*	*p*-value
Negative	0.342	0.063	0.331	0.060	0.188	0.430
Neutral	0.609	0.058	0.618	0.051	−0.169	0.487
Positive	0.049	0.016	0.051	0.017	−0.148	0.526

### 4.2 Linguistic analysis of synthetic data

Statistical analysis of the synthetic dataset revealed substantial linguistic differences between deceptive and truthful participants. Identical to the linguistic analysis of the military exercise dataset, Welch’s *t*-tests were conducted for all POS categories and total word count. As shown in Table 5, 26 linguistic features reached nominal significance (*p* < 0.05) prior to correction for multiple comparisons. Following B-H FDR correction, 25 features remained statistically significant, whereas 21 remained significant following the more conservative Bonferroni correction. Total word count, which reached nominal significance (*p* = 0.0369), did not remain significant after FDR correction (adjusted *p* = 0.0611) or Bonferroni correction (adjusted *p* = 1.000). The persistence of significant differences across a large number of linguistic features after both correction procedures indicates substantial separation between the synthetic truthful and deceptive classes. These widespread differences likely reflect systematic linguistic patterns introduced during synthetic data generation and should not be interpreted as evidence that the same features reliably distinguish deception from truthfulness in naturally occurring human language.

**Table 5:** Summary of *p*-values following a series of Welch’s *t*-tests on all 44 POS features extracted from the synthetic data, including FDR and Bonferroni corrections.

Synthetic data features	*p*-value	B-H FDR correction	Bonferroni correction
Tos	4.78E-38	2.06E-36	2.06E-36
Singular nouns	**5.13E-33**	**1.10E-31**	**2.21E-31**
Base form verbs	**1.64E-29**	**2.35E-28**	**7.05E-28**
All nouns	**3.05E-19**	**3.28E-18**	**1.31E-17**
Regular determiners	**2.05E-18**	**1.76E-17**	**8.82E-17**
All determiners	**1.65E-14**	**1.18E-13**	**7.10E-13**
Plural nouns	**2.83E-09**	**1.74E-08**	**1.22E-07**
Past participle verbs	**1.16E-08**	**6.24E-08**	**4.99E-07**
Whadverbs	**2.23E-07**	**1.07E-06**	**9.59E-06**
Modal verbs	**3.08E-07**	**1.32E-06**	**1.32E-05**
Prepositions	**3.49E-07**	**1.35E-06**	**1.50E-05**
Past tense verbs	**3.76E-07**	**1.35E-06**	**1.62E-05**
Whpronouns	**5.11E-06**	**1.69E-05**	**0.00021973**
Regular adjectives	**2.42E-05**	**7.43E-05**	**0.0010406**
Cardinal numbers	**2.84E-05**	**8.14E-05**	**0.0012212**
Gerunds	**6.73E-05**	**0.000180869**	**0.0028939**
All verbs	**8.02E-05**	**0.000202859**	**0.0034486**
Comparative adverbs	**0.000138236**	**0.00033023**	**0.005944148**
All adjectives	**0.00014839**	**0.00033583**	**0.00638077**
Singular proper nouns	**0.000169525**	**0.000364479**	**0.007289575**
Superlative adverbs	**0.000753513**	**0.001542908**	**0.032401059**
Coordinating conjunctions	**0.001474437**	**0.002881854**	0.063400791
Possessive pronouns	**0.00174757**	**0.003267196**	0.07514551
Singular 3rd person verbs	**0.002295301**	**0.004112414**	0.098697943
Superlative adjectives	**0.016982618**	**0.029210103**	0.730252574
Total words	**0.036938419**	0.061090462	1
Comparative adjectives	0.064324407	0.102442574	1
Personal pronouns	0.067968096	0.104379576	1
All adverbs	0.181147924	0.266110553	1
Singular Non3rd person verbs	0.185658525	0.266110553	1
All conjunctions/prepositions	0.249894117	0.342483252	1
All pronouns	0.255532607	0.342483252	1
Particles	0.262835984	0.342483252	1
Predeterminers	0.315633026	0.390434073	1
Symbols	0.317795176	0.390434073	1
Exestential Theres	0.328434797	0.392297119	1
Others	0.413338648	0.480366537	1
Possessive endings	0.413338648	0.467725312	1
Interjections	0.478231556	0.527280946	1
Possessive whpronouns	0.563586869	0.605855884	1
Plural proper nouns	0.704813079	0.739194205	1
Whdeterminers	0.862613971	0.883152399	1
Regular adverbs	0.9004577	0.9004577	1

Bold values indicate statistically significant results (*p* < 0.05) in the corresponding unadjusted or corrected test column.

Effect-size analysis narrowed the wide range of features deemed significant from the Welch’s *t*-test. As shown in Figure 2, Cohen’s *d* effect size tests revealed high effects for singular nouns (*d* = −0.79) and tos—typically functioning as infinitive markers (e.g., “to go”) or directional prepositions (e.g., “to the camp”)—(*d* = 0.85). This test also uncovered moderate effects for determiners, as a broad category (*d* = −0.49), regular determiners (*d* = −0.56), nouns, also as a broad category of all forms of nouns (*d* = −0.58), and regular verbs (*d* = 0.74). While reaching statistical significance, plural nouns, past participle verbs, whadverbs, and modal verbs (|*d* ≈ 0.30|) all constitute low effect size.

**Figure 2:**
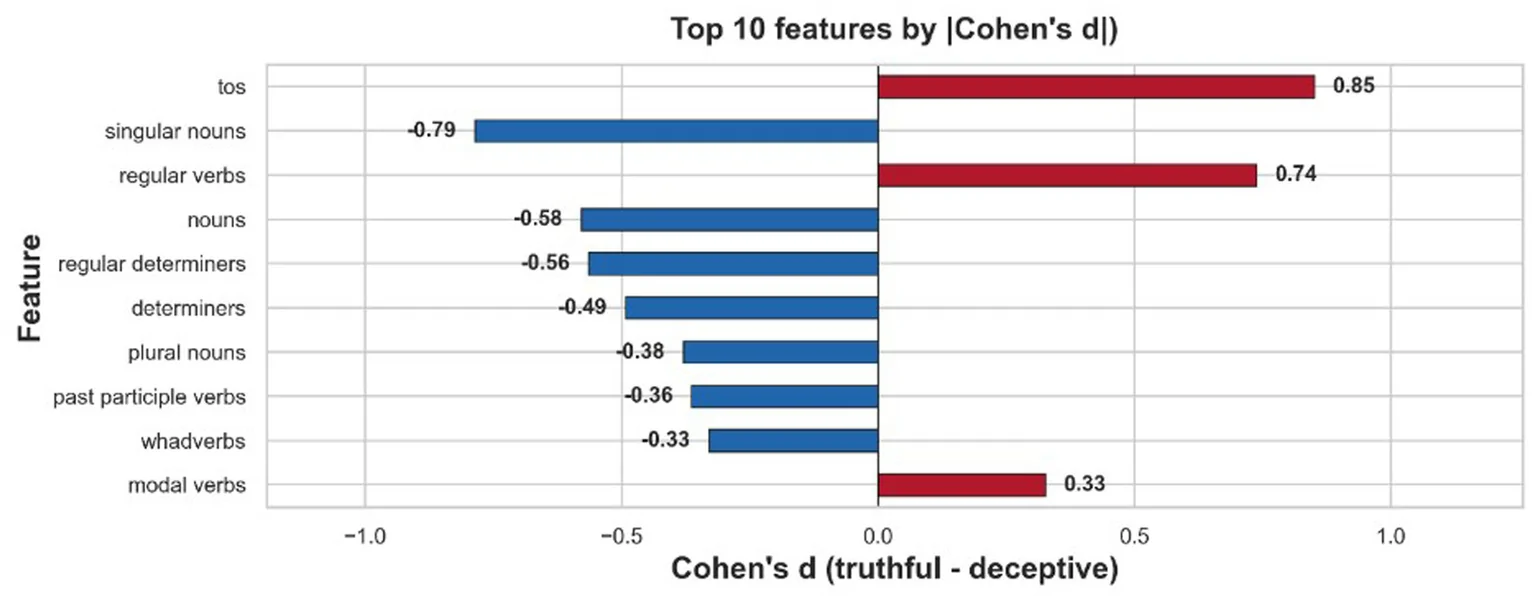
Cohen’s *d* effect sizes for the 10 most discriminative POS categories found in the synthetic dataset. Large absolute values (|*d*| ≈ 0.8) indicate substantial differences between classes. Singular nouns and “tos,” often used as infinitive markers or prepositions, were both found to have large effect sizes (|Cohen’s *d*| ≈ 0.8). Tos were more prevalent in deceptive responses, while singular nouns more prevalent in truthful responses.

Sentiment analysis in the synthetic dataset revealed significant differences between deceptive and truthful responses across all three sentiment dimensions. As shown in Table 6, deceptive responses exhibited significantly greater negative sentiment (mean = 0.290, SD = 0.088) than truthful responses (mean = 0.257, SD = 0.111; *p* = 3.02E-7), although the magnitude of this difference was small (*d* = 0.326). Neutral sentiment was also significantly higher in deceptive responses (mean = 0.607, SD = 0.055) than truthful responses (mean = 0.589, SD = 0.056; *p* = 6.18E-7), similarly yielding a small effect size (*d* = 0.317). In contrast, positive sentiment was significantly lower in deceptive responses (mean = 0.103, SD = 0.045) than truthful responses (mean = 0.154, SD = 0.086; *p* = 1.11E-28) and demonstrated the largest effect among the three sentiment dimensions (*d* = −0.733). Collectively, these findings indicate that the synthetic truthful and deceptive classes differed systematically in emotional tone, with comparatively small differences in negative and neutral sentiment but a substantially larger separation in positive sentiment. This pattern provides additional evidence that the synthetic dataset contains pronounced class-specific linguistic characteristics that may facilitate classification but may not generalize to naturally occurring, high-stakes deception.

**Table 6:** Sentiment analysis summary for deceptive and non-deceptive synthetic dataset responses, including group means, standard deviations, Cohen’s *d*, and *p*-values across negative, neutral, and positive polarity.

Sentiment	Deceptive mean	Deceptive SD	Truthful mean	Truthful SD	Cohen’s *d*	*p*-value
Negative	0.290	0.088	0.257	0.111	0.326	3.02E-07
Neutral	0.607	0.055	0.589	0.056	0.317	6.18E-07
Positive	0.103	0.045	0.154	0.086	−0.733	1.11E-28

### 4.3 Supervised models—synthetic dataset (*N* = 300)

Model performance on the synthetic dataset was evaluated using a held-out test set composed of 150 deceptive and 150 non-deceptive responses. Performance metrics for the four supervised machine learning models are presented in Table 7. Among these models, the logistic regression classifier performed best, with an F1-score of 0.930, precision of 0.927, recall of 0.933, accuracy of 0.930, and ROC AUC of 0.978. SVM also performed well, with an F1-score of 0.908, precision of 0.896, recall of 0.920, accuracy of 0.907, and ROC AUC of 0.974. The random forest model achieved an F1-score of 0.887 and accuracy of 0.887, while naïve Bayes produced an F1-score of 0.878 and accuracy of 0.880. FranklinAsk™ performed marginally worse than the supervised models on this dataset, producing an F1-score, precision, recall, accuracy of 0.733 and an AUC of 0.734.

**Table 7:** Performance metrics for all supervised ML models and FranklinAsk™ on the synthetic test dataset.

Synthetic data	F-1 score	Precision	Recall	Accuracy	ROC AUC
Naïve bayes	0.878[CI: 0.827–0.903]	0.890[CI: 0.828–0.906]	0.867[CI: 0.827–0.903]	0.880[CI: 0.827–0.903]	0.938[CI: 0.920–0.967]
Random forest	0.887[CI: 0.847–0.920]	0.882[CI: 0.847–0.9209]	0.893[CI: 0.847–0.920]	0.887[CI: 0.847–0.920]	0.957[CI: 0.940–0.978]
Logistic regression	0.930[CI: 0.890–0.950]	0.927[CI: 0.890–0.952]	0.933[CI: 0.890–0.950]	0.930[CI: 0.890–0.950]	0.978[CI: 0.951–0.986]
SVM	0.908[CI: 0.892–0.950]	0.896[CI: 0.892–0.952]	0.920[CI: 0.892–0.950]	0.907[CI: 0.892–0.950]	0.974[CI: 0.962–0.987]
FranklinAsk	0.733[CI: 0.674–0.787]	0.733[CI: 0.660–0.802]	0.733[CI: 0.662–0.804]	0.733[CI: 0.683–0.783]	0.734[CI: 0.680–0.785]

Metrics include F1-score, precision, recall, accuracy, and ROC AUC with 95% confidence intervals.

To further illustrate classification behavior, confusion matrices for all four supervised models are shown in Figures 3A–D. These matrices highlight model-specific tendencies, such as naïve Bayes producing a larger proportion of false negatives and random forest generating comparatively more false positives. The FranklinAsk system, evaluated on the same held-out test data, exhibited moderate performance (Figure 3E), revealing significantly more false positives and false negatives, consistent with its knowledge-driven reasoning approach rather than statistical learning.

**Figure 3:**
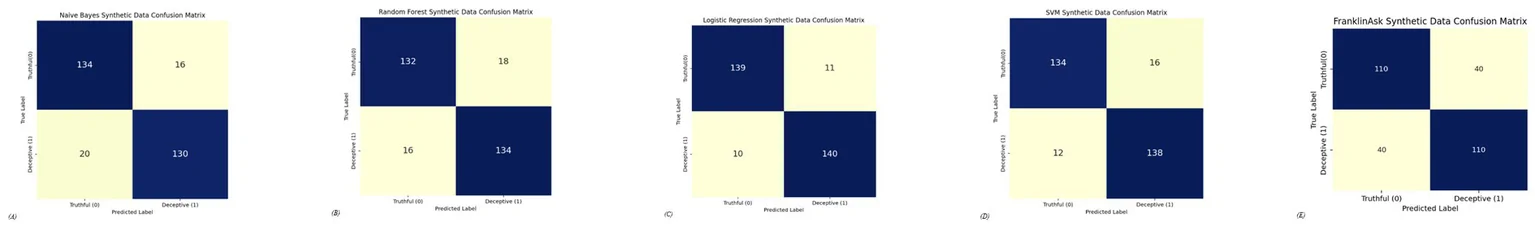
**(A–D)** Confusion matrices for naïve Bayes, random forest, logistic regression, and SVM on synthetic test data. **(E)** Confusion matrix for FranklinAsk™ evaluated on the same partition. Darker shades indicate more frequent classification outcomes.

### 4.4 Supervised models—military exercise data (*N* = 76)

All supervised machine learning models were subsequently evaluated on the complete military dataset to assess generalizability. As shown in Table 8, model performance dropped sharply across all metrics when evaluated on real-world data. Logistic regression achieved an F1-score of 0.456, a precision of 0.367, a recall of 0.600, an accuracy of 0.434, and an ROC AUC of 0.417. SVM performed similarly, with an F1-score of 0.400, precision of 0.350, recall of 0.467, and accuracy of 0.447. Naïve Bayes performed the poorest among the supervised models, producing an F1-score of 0.299, precision of 0.270, recall of 0.333, accuracy of 0.382, and ROC AUC of 0.376. This weak performance is reflected in Figure 4A, where the Naïve Bayes confusion matrix shows substantial misclassification across both classes.

**Table 8:** Performance metrics for supervised ML models and FranklinAsk™ when applied to the real-world military dataset with 95% confidence intervals.

Military exercise testing	F-1 score	Precision	Recall	Accuracy	ROC AUC
Naïve bayes	0.299[CI: 0.111–0.393]	0.270[CI: 0.115, 0.435]	0.333[CI: 0.100–0.393]	0.382[CI: 0.329, 0.540]	0.357[CI: 0.242, 0.488]
Random forest	0.473[CI: 0.357, 0.615]	0.349[CI: 0.250–0.500]	**0.733**[CI: 0.562, 0.885]	0.355[CI: 0.303, 0.513]	0.376[CI: 0.329, 0.597]
Logistic regression	0.456[CI: 0.282, 0.541]	0.367[CI: 0.204, 0.460]	0.600[CI: 0.389, 0.735]	0.434[CI: 0.263, 0.474]	0.417[CI: 0.238, 0.500]
SVM	0.400[CI: 0.222, 0.494]	0.350[CI: 0.182, 0.462]	0.467[CI: 0.259, 0.607]	0.447[CI: 0.302, 0.513]	0.407[CI: 0.217, 0.467]
FranklinAsk	0.519[CI: 0.400–0.648]	0.412[CI: 0.278–0.527]	0.700[CI: 0.645–0.933]	0.487[CI: 0.342–0.553]	0.487[CI: 0.424–0.675]

FranklinAsk™ exhibits the highest F1-score, precision, and accuracy among all evaluated methods.

Although the Random Forest achieved the highest recall (0.733) among the supervised models, its precision dropped to 0.349, reflecting its tendency to classify most responses as deceptive. This imbalance is reflected in Figure 4B, which shows almost no true-negative predictions, resulting in a substantial false-positive rate. Figures 4C,D show that logistic regression and SVM also produced substantial classification errors.

**Figure 4:**
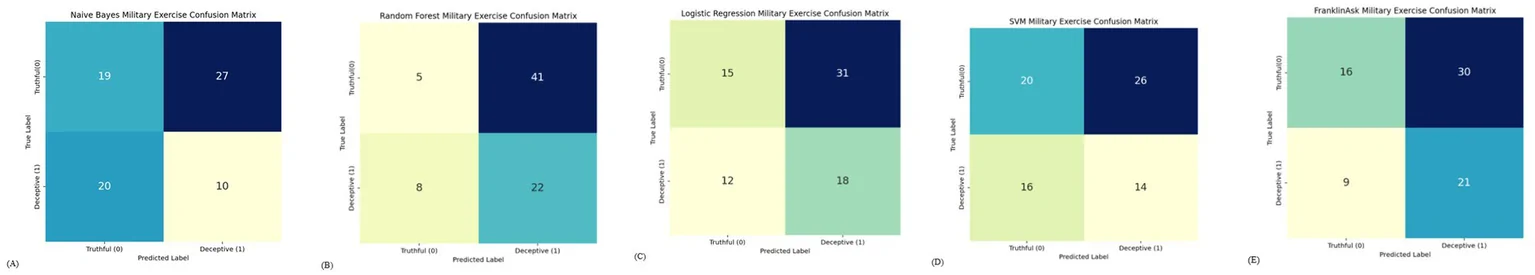
**(A–D)** Confusion matrices for naïve Bayes, random forest, logistic regression, and SVM evaluated on the military dataset. **(E)** Confusion matrix for FranklinAsk™ on the same dataset. Models show distinct error patterns, with supervised methods degrading substantially in real-world conditions. Confusion matrices are displayed with rows = true labels and columns = predicted labels.

In contrast to the supervised models, FranklinAsk™ exhibited a different profile on the military dataset. FranklinAsk™ achieved an F1-score of 0.519, precision of 0.412, recall of 0.700, and accuracy of 0.487. These metrics are reflected in Figure 4E, showing that FranklinAsk™ produced fewer false negatives. FranklinAsk™ produced the highest point estimates for F1-score, precision, and accuracy, whereas Random Forest produced the highest recall. Given the military dataset size (*n* = 76), these differences are descriptive and should not be interpreted as demonstrating statistical superiority.

### 4.5 Cross-study comparison and domain effects

To contextualize the performance of the models used in this study, results were compared with those reported by Brzic et al. (2023), who evaluated the same four supervised algorithms using LIWC-22 features on two fake-news datasets. Table 9 summarizes this comparison. In the synthetic dataset used in the present study, models achieved higher accuracy than those in Brzic et al. work; for instance, the logistic regression achieved an accuracy of 93.0% compared with 78.02 and 74.62% in Brzic et al. climate-change and COVID-19 datasets, respectively. However, when the models were applied to the real-world military dataset, accuracies dropped dramatically, with SVM reaching only 44.70% and random forest dropping to 35.5%.

**Table 9:** Comparison of classification accuracies between the present study and Brzic et al. (2023) across two external fake-news datasets (climate change and COVID-19). Results illustrate substantial cross-domain performance variability.

Accuracies using LIWC-22	Our models –synthetic dataset	Our models -military exercise dataset	Brzic et al. (2023) -climate change dataset	Brzic et al. (2023) -COVID-19 dataset
Logistic regression	**93.00%**	43.42%	78.02%	74.62%
Random forest	**88.70%**	35.53%	78.05%	69.98%
Naïve bayes	**88.00%**	38.16%	76.47%	74.58%
SVM	**90.70%**	44.70%	77.24%	73.32%

Bold values indicate the highest classification accuracy for each model across the compared datasets.

This discrepancy reinforces the role of domain characteristics in model performance. Structured fake-news datasets, such as those used by Brzic et al., tend to use longer, more uniform language and do not reflect the spontaneity or cognitive stress associated with deception in operational settings. By contrast, the military dataset used in this study captures spontaneous, stress-induced language, which presents a substantially more challenging environment for supervised classification models. These results collectively underscore the importance of domain-appropriate data and demonstrate that high performance in synthetic or low-stakes environments may not translate to real-world deception detection.

## 5 Discussion

The purpose of this study was to evaluate linguistic markers of deception and compare the performance of supervised ML methods and a RAG system when applied to both synthetic and real-world deception datasets. The findings reveal three central observations. First, the real-world dataset provided limited evidence of linguistic differences between deceptive and truthful speech after correction for multiple comparisons. Cardinal numbers and particles reached nominal significance, but neither remained statistically significant after FDR or Bonferroni correction. Second, supervised ML models performed exceptionally well on large, balanced synthetic datasets, yet their performance deteriorated sharply when applied to high-stakes, spontaneous responses. Third, FranklinAsk™ produced the highest point estimates for accuracy, precision, and F1-score in the real-world dataset, whereas Random Forest produced the highest recall. Because the approaches differed in both architecture and information available at inference, and because the military sample was small, these descriptive differences do not establish superiority or isolate an effect of retrieval augmentation.

The linguistic analysis of the real-world dataset supports prior research showing that deception often produces subtle and context-sensitive changes in language. Although a wide range of linguistic features were evaluated, two reached nominal significance before multiplicity correction and both exhibited moderate unadjusted effect sizes (*d* = 0.55); neither remained statistically significant after FDR or Bonferroni correction. The elevated use of cardinal numbers and particles in deceptive responses may reflect increased cognitive load, as deceptive individuals attempt to construct plausible narratives, maintain internal consistency, or anchor fabricated events with numerical or structural cues. Given the large number of linguistic features tested relative to sample size, the observed nominally significant effects may represent false-positive findings. Replication in larger datasets is required before concluding that cardinal numbers or particles constitute robust markers of deception. This interpretation aligns with the cognitive-load literature, which suggests that deception requires additional mental resources, thereby altering syntactic planning and lexical selection. Other features showed small effects but did not reach significance, reinforcing the conclusion that no single linguistic cue reliably signals deception in spontaneous speech. The absence of meaningful differences in sentiment further confirms that emotional tone alone is a weak predictor of deceptive intent, especially in structured or high-stakes assessment scenarios.

In contrast to the real-world dataset, the synthetic dataset exhibited widespread linguistic separation between deceptive and truthful classes. Welch’s *t*-tests revealed that approximately two-thirds of POS categories differed significantly, and several features demonstrated moderate to large effect sizes, including various forms of nouns, determiners, verbs, and infinitive markers. Unlike the military dataset, sentiment also significantly differentiated classes, including a moderately large effect for positive sentiment favoring truthful responses. These findings suggest that the synthetic dataset appears to encode idealized, stylized distinctions between truthful and deceptive language rather than subtle, ecologically valid linguistic variation.

Striking differences exist between the statistical analysis of these two datasets. In the military dataset, two linguistic features reached nominal significance before multiplicity correction, each with a moderate unadjusted effect size; neither remained statistically significant after FDR or Bonferroni correction. In the synthetic dataset, 26 features were statistically significant, several with moderate or large effects. This discrepancy illustrates how generative models may exaggerate linguistic cues of deception, creating boundaries that are far more noticeable than those observed in high-stakes, stress-induced speech. The superior performance of the supervised ML models is likely attributable to the substantial linguistic separations observed in the synthetic dataset, making the classification a less complex task.

Furthermore, the substantial performance difference between synthetic and military datasets suggests that synthetic text generation may introduce linguistic regularities that are easier to classify than naturally occurring deceptive behavior, with synthetic performance at ~0.73–0.93 accuracy versus military performance at ~0.35–0.49 accuracy. Because synthetic responses were generated using explicit truthful-versus-deceptive instructions, some language patterns may reflect LLM representations or stereotypes of deception rather than authentic deceptive communication. Consequently, results obtained on synthetic datasets should not be interpreted as directly transferable to operational settings.

The supervised ML models trained on the synthetic dataset performed remarkably well, with the logistic regression achieving an F1-score of 0.930 and a ROC AUC of 0.978. SVM produced similarly strong metrics, while random forest and naïve Bayes performed slightly less effectively but still achieved accuracies close to 90%. FranklinAsk produced lower point estimates than the supervised models on the synthetic dataset with an F1-score of 0.733. Because synthetic responses incorporate no domain-specific deception literature, the hybrid-RAG system lacks evidence to ground its judgments, reducing performance relative to models trained directly on synthetic statistics. Overall, these results demonstrate that LIWC-based features, when paired with balanced and abundant data, can support reliable deception classification. However, the dramatic decline in performance on the real-world dataset underscores a major limitation of using synthetic or low-stakes text to train deception-detection models. When confronted with the short, variable, and cognitively stressed responses present in the military exercise data, each model exhibited substantial decreases in F1-score, precision, and ROC AUC. For example, logistic regression, which achieved 93.0% accuracy on synthetic data, achieved only 43.4% accuracy on the real-world dataset, and random forest dropped from 88.7 to 35.5%. FranklinAsk™ exhibited the least dramatic decline in performance, dropping from an accuracy of 73.3 to 48.7%. These discrepancies demonstrate that synthetic data alone cannot fully capture the linguistic complexity and variability present in high-stakes deception, and that model generalization remains a significant challenge.

Performance patterns on the real-world dataset differed across metrics. FranklinAsk™ achieved the highest point estimates for F1-score (0.519), precision (0.412), and accuracy (0.487), whereas Random Forest achieved the highest recall (0.733), compared with 0.700 for FranklinAsk™. These point-estimate differences should not be interpreted as statistical superiority. FranklinAsk™ incorporated external domain knowledge and foundation-model reasoning capabilities unavailable to the LIWC-based supervised classifiers. Consequently, the observed performance differences cannot be attributed specifically to retrieval augmentation or model architecture alone. They may reflect access to curated deception literature, prompt design, base-model reasoning, and different input representations. Controlled ablation studies are needed to isolate the contribution of each component. The present results therefore motivate further evaluation of knowledge-grounded systems rather than demonstrating an inherent advantage over conventional machine-learning approaches.

At the same time, several limitations must be acknowledged. The military dataset, while ecologically valuable, comprised only 76 participants, including 30 deceptive participants, with each participant providing eight brief open-ended responses. This sample size limits the precision of classifier-performance estimates and the statistical power of linguistic analyses. Accordingly, model rankings based on point estimates are descriptive rather than evidence of statistically reliable differences, and generalization beyond this exercise should be approached cautiously. Furthermore, synthetic text, although helpful in mitigating class imbalance, may oversimplify or distort natural patterns of deception, reducing its effectiveness as a training surrogate. Regarding FranklinAsk, the relative contribution of domain knowledge, prompt design, retrieval mechanisms, and base-model reasoning remains an open question that requires controlled ablation studies. Future work should address these limitations by collecting larger, naturalistic datasets, exploring multimodal indicators such as acoustic and physiological signals, and expanding the knowledge base used by the RAG system to incorporate cross-domain deception studies.

Despite these limitations, the present findings contribute to the growing literature on NLP-based deception detection by offering a rare comparison of supervised ML and RAG methodologies across both synthetic and real-world datasets. The results show that high performance on synthetic datasets does not reliably predict real-world effectiveness, and that linguistic cues of deception are subtle, context-dependent, and easily overshadowed by response brevity or stress. They also motivate further evaluation of knowledge-grounded AI systems, such as FranklinAsk™, in settings characterized by limited or noisy training data. Larger independent datasets are required before drawing conclusions about comparative or operational performance. Together, these findings underscore the need for hybrid approaches that combine linguistic feature analysis, machine learning, and knowledge-augmented reasoning to better capture the complexity of deception in naturalistic settings.

## Data Availability

The raw data supporting the conclusions of this article will be made available by the authors, without undue reservation.

## Author disclaimer

The views expressed in this article are those of the authors and do not necessarily reflect the official policy or position of the Director of National Intelligence, the Department of War, or the U. S. Government. The United States Government retains a royalty-free, nonexclusive, and irrevocable license to reproduce, translate, publish, and use this manuscript (32CFR32.36).
