# Supplementary material for: Leveraging natural language processing, machine learning, and a knowledge-grounded language model to detect linguistic markers of deception
Source: Front Artif Intell. 2026 Oct 28;9:1965099. doi: 10.3389/frai.2026.1965099 (PMC13752212; doi:10.3389/frai.2026.1965099)
Supplement: Supplementary file 1 [file Data_Sheet_1.docx]

# **Appendix A. FranklinAsk^TM^ Methodology and Experimental Configuration**

## **Model Configuration**

FranklinAsk was configured using Claude Haiku 4.5, model version haiku-4-5-20251001. Model temperature was fixed at 0 for all analyses to promote deterministic and reproducible responses. The top_p and top_k parameters were not explicitly specified and therefore remained at their system default values.

The FranklinAsk methodology consisted of three sequential stages: 1.) screening the candidate literature for relevance to linguistic deception detection, 2.) extracting deception-related linguistic characteristics from the relevant literature, and 3.) applying the resulting knowledge context to participant responses from the dataset to estimate the likelihood of deception.

## **Literature Screening and Knowledge-Source Selection**

Twenty-one documents were initially provided to FranklinAsk as candidate knowledge sources. To identify documents containing information directly relevant to linguistic deception detection, each document was evaluated using the following prompt:

*“For each document, indicate Yes or No, whether the document provides a detailed description of word choices, sentence structure, cognitive and emotional indicators, and other linguistic characteristics that would indicate possible deception. Also, provide a short justification for each.”*

FranklinAsk classified 9 of the 21 documents as relevant to the linguistic identification of deception. The remaining 12 documents were excluded from the augmented knowledge used for deception classification because they focused primarily on general NLP methods, semantic parsing, sentiment analysis, model training, truthfulness of LLM outputs, or other topics without providing substantive linguistic indicators of human deception.

The nine documents retained as relevant were:

*Real-World Deception and the Impact of Severity,* Fuller et al. (2015) [20]

*Analysing Deception in Witness Memory through Linguistic Styles in Spontaneous Language,* Solà-Sales et al. (2023) [21]

*Detecting Deception through Linguistic Cues: From Reality Monitoring to Natural Language Processing,* Locante et al. (2025) [1]

*The Linguistic Correlates of Conversational Deception: Comparing Natural Language Processing Technologies,* Duran et al. (2010*)* [22]

*Building and Benchmarking the Motivated Deception Corpus: Improving the Quality of Deceptive Text through Gaming,* Barsever et al. (2023) [23]

*BERTective: Language Models and Contextual Information for Deception* *Detection* Fornaciari et al. (2021) [24]

*Automatic Detection of Verbal Deception*, Fitzpatrick et al. (2015) [25]

*Computational Measures of Deceptive Language*, Tomas et al. (2022) [26]

*Truth or Lie: Exploring the Language of Deception,* Sarzynska-Wawer et al. (2023) [15]

The retained literature included research addressing linguistic features such as lexical diversity, pronoun use, negation, sentence length and complexity, parts-of-speech patterns, temporal and spatial information, cognitive-load markers, affective language, uncertainty, distancing strategies, contextual details, speech disfluencies, and other verbal characteristics that have been investigated in relation to deception.

## **Extraction of Linguistic Deception Criteria**

Following the relevance-screening stage, each of the nine retained documents was queried independently to extract the linguistic characteristics potentially associated with deception. The following prompt was used for each source:

*“Provide a detailed description of word choices, sentence structure, cognitive and emotional indicators, and other linguistic characteristics that would indicate possible deception.”*

The responses generated from this extraction prompt constituted the document-level synopses subsequently concatenated into the augmented knowledge context. The resulting outputs were then used to construct the augmented knowledge context supplied to FranklinAsk for evaluation of the interview question responses. This approach was intended to constrain the subsequent deception assessment to linguistic criteria derived from the selected deception literature rather than relying exclusively on the language model's general parametric knowledge.

Because the relevant literature-derived information could be accommodated within the available model context, the experimental implementation used the aggregated knowledge context during inference rather than requiring dynamic retrieval of individual document chunks for each participant. Thus, while FranklinAsk is based on a retrieval-augmented architecture, the configuration evaluated in this study is more precisely characterized as knowledge-grounded inference using a preassembled augmented context.

## **Synthetic and Military Dataset Evaluation**

For the synthetic and military datasets, the interview responses associated with each participant were evaluated against the aggregated linguistic deception criteria derived from the nine relevant sources. FranklinAsk was instructed to estimate each participant's likelihood of deception using the following prompt:

*“Based strictly on the criteria provided on linguistic indicators of deception and truthfulness, can you rate on a scale of 1 to 10 (10 being most likely) this subject's likelihood of being deceptive with explanations?”*

FranklinAsk therefore generated a 1-to-10 deception-likelihood rating, with higher values representing greater estimated likelihood of deception. The prompt explicitly instructed the system to base its judgment on the literature-derived linguistic criteria supplied through the augmented knowledge context.

This process was applied consistently across participants using the same model configuration, augmented knowledge, and inference prompt. A temperature of 0 was maintained throughout the evaluation.

## **Reproducibility and Interpretation**

The FranklinAsk analysis should be interpreted as a knowledge-grounded language-model classification approach rather than as an equivalent implementation of the supervised LIWC-based classifiers evaluated elsewhere in the study. FranklinAsk had access to a curated body of domain-specific literature and foundation-model reasoning capabilities that were not available to the supervised ML models. Consequently, differences in performance between FranklinAsk and the ML classifiers cannot be attributed specifically to retrieval augmentation, model architecture, or any single system component.

The experimental configuration was held constant across both synthetic and military datasets with respect to the language model version, temperature, augmented knowledge context, and classification prompt. Specifically, the implementation used Claude Haiku 4.5 (haiku-4-5-20251001) with temperature set to 0; top_p and top_k were left unspecified and therefore used their default settings.
