# Supplementary material for: Leveraging natural language processing, machine learning, and a knowledge-grounded language model to detect linguistic markers of deception
Source: Front Artif Intell. 2026 Oct 28;9:1965099. doi: 10.3389/frai.2026.1965099 (PMC13752212; doi:10.3389/frai.2026.1965099)
Supplement: Supplementary file 2 [file Data_Sheet_2.docx]

Fuller, C. M., Biros, D. P., Twitchell, D. P., and Wilson, R. L. (2015). Real-world deception and the impact of severity. *J. Comput. Inf. Syst.* 55, 59–67. doi: 10.1080/08874417.2015.11645757

Solà-Sales, S., Alzetta, C., Moret-Tatay, C., and Dell’Orletta, F. (2023). Analysing deception in witness memory through linguistic styles in spontaneous language. *Brain Sci.* 13. doi: 10.3390/brainsci13020317

Loconte, R., Battaglini, C., Maldera, S., Pietrini, P., Sartori, G., Navarin, N., et al. (2025). Detecting deception through linguistic cues: from reality monitoring to natural language processing. *J. Lang. Soc. Psychol.* 44, 523–552. doi: 10.1177/0261927X251316883

Duran, N. D., Hall, C., McCarthy, P. M., & McNamara, D. S. (2010). The linguistic correlates of conversational deception: Comparing natural language processing technologies. Applied Psycholinguistics, 31(3), 439–462. <https://doi.org/10.1017/S0142716410000068>

Barsever, D., Steyvers, M., and Neftci, E. (2023). Building and benchmarking the moti­vated deception corpus: improving the quality of deceptive text through gaming. *Behav. Res. Methods* 55, 4478–4488. doi: 10.3758/s13428-022-02028-7

Fornaciari, T., Poesio, M., Bianchi, F., and Hovy, D. (2021) *BERTective: Language Models and Contextual Information for Deception Detection*

Fitzpatrick, E., Bachenko, J.Llc, L., and Fornaciari, T. (2015) *Automatic Detection of Verbal Deception*. Available online at: <http://store.morganclaypool.com>

Tomas, F., Dodier, O., and Demarchi, S. (2022). Computational measures of deceptive lan­guage: prospects and issues. *Front. Commun.* 7:792378. doi: 10.3389/fcomm.2022.792378

Sarzynska-Wawer, J., Pawlak, A., Szymanowska, J., Hanusz, K., and Wawer, A. (2023). Truth or lie: exploring the language of deception. *PLoS One* 18:e0281179. doi: 10.1371/ journal.pone.0281179
